# Key Matrix Remodeling Enzymes: Functions and Targeting in Cancer

**DOI:** 10.3390/cancers13061441

**Published:** 2021-03-22

**Authors:** Zoi Piperigkou, Konstantina Kyriakopoulou, Christos Koutsakis, Stylianos Mastronikolis, Nikos K. Karamanos

**Affiliations:** 1 Biochemistry, Biochemical Analysis and Matrix Pathobiology Research Group, Laboratory of Biochemistry, Department of Chemistry, University of Patras, 265 04 Patras, Greece; k.kyriakopoulou@upnet.gr (K.K.); ckoutsakis@upatras.gr (C.K.); 2 Foundation for Research and Technology-Hellas (FORTH)/Institute of Chemical Engineering Sciences (ICE-HT), 265 04 Patras, Greece; 3 Faculty of Medicine, University of Crete, 700 13 Heraklion, Greece; med3541@edu.med.uoc.gr

**Keywords:** extracellular matrix, cancer, matrix metalloproteinases, hyaluronidases, heparanase, plasminogen activators, cathepsins

## Abstract

**Simple Summary:**

Proteolytic enzymes, such as matrix metalloproteinases, plasminogen activators and cathepsins, as well as non-proteolytic enzymatic partners, such as heparanase and hyaluron-idases, play key roles in the propagation and metastatic potential of cancer cells. This article aims to revisit the main functions of major matrix remodeling molecules and their effects in cancer meta-static potential. Moreover, the epigenetic regulation mechanisms of these molecules are discussed, in addition to recent advances in their pharmacological targeting. Finally, novel data from ongoing clinical trials on several cancer types are also provided. Overall, this review delves into the im-portance of matrix remodeling partners in cancer metastasis and explores their targeting as a promising therapeutic option for cancer management.

**Abstract:**

Tissue functionality and integrity demand continuous changes in distribution of major components in the extracellular matrices (ECMs) under normal conditions aiming tissue homeostasis. Major matrix degrading proteolytic enzymes are matrix metalloproteinases (MMPs), plasminogen activators, atypical proteases such as intracellular cathepsins and glycolytic enzymes including heparanase and hyaluronidases. Matrix proteases evoke epithelial-to-mesenchymal transition (EMT) and regulate ECM turnover under normal procedures as well as cancer cell phenotype, motility, invasion, autophagy, angiogenesis and exosome formation through vital signaling cascades. ECM remodeling is also achieved by glycolytic enzymes that are essential for cancer cell survival, proliferation and tumor progression. In this article, the types of major matrix remodeling enzymes, their effects in cancer initiation, propagation and progression as well as their pharmacological targeting and ongoing clinical trials are presented and critically discussed.

## 1. Introduction

Matrix degradation is a fine-tuned process that coexists with the production of newly formed ECM molecules. Tissue integrity is achieved through ECM replacement that happens thanks to the actions of matrix-degrading enzymes, such as matrix metalloproteinases (MMPs) and their endogenous inhibitors (TIMPs), the adamalysin group (ADAMs and ADAMTS), plasminogen activation system components, cathepsins and glycolytic enzymes, such as heparanase (HPSE) and hyaluronidases (HYALs) that cleave heparan sulfate (HS)/heparin chains on proteoglycans (PGs) and hyaluronan (HA) [1,2]. ECM serine proteases also involve elastase, dipeptidyl peptidase IV (DPPIV) and tissue kallikrein unique roles in matrix proteolysis and have been associated with cancer progression [3,4,5].

MMPs are the principal catabolic matrix endopeptidases that have been associated with a variety of normal conditions, including wound healing, immune response, differentiation and tissue homeostasis, as well as with several diseases as osteoarthritis, neuroinflammation, atherosclerosis and cancer [6]. A total of twenty-three MMP members have been identified in the human genome that are divided into secreted and membrane bound MMPs. Depending on substrate specificity, MMPs are classified into matrilysins, gelatinases, furin-activated, collagenases, stromelysins, and other MMPs [7,8]. MMPs are considered mediators of the alterations observed in the tumor microenvironment during cancer progression since they promote epithelial-to-mesenchymal transition (EMT), cancer cell signaling, migration, invasion, autophagy and angiogenesis that facilitate tumor progression and metastasis [9].

The plasminogen activation (PA) system is a proteolytic system responsible for the conversion of plasminogen (PLG) to plasmin and subsequent activation of fibrinolysis [10]. The key role of the PA system in a plethora of functions including ECM degradation, fibrinolysis, cell migration and angiogenesis has linked its components to tumorigenesis. Thus, the plasminogen activation proteolytic cascade emerges as an important system that acts towards promotion of metastasis.

Cathepsins are a large family of proteases with broad specificity which are localized primarily in lysosomes and have pivotal roles in various normal and pathological processes, such as immune response, cell homeostasis, neurodegenerative diseases and cardiovascular disorders [11]. In cancer, where ECM degradation is necessary for metastasis, cathepsins facilitate ECM remodeling and disruption of cell-cell junctions to promote migration and invasion, while they also advance angiogenesis and chemoresistance [12]. 

Aside from the proteolytic matrix partners, two enzyme families with glycosidase activity, HPSE and HYALs, also play a key role in ECM remodeling. The high expression of HPSE in most malignancies exemplifies its role in cancer propagation, while its activity is closely linked to tumor progression and metastasis through the means of angiogenesis, autophagy, EMT and exosome biogenesis [13]. Because of these implications, HPSE inhibition constitutes an attractive pharmacological target for the development of cancer therapies. HYALs are endo-β-N-acetylglucosaminidases that degrade HA into smaller fragments. Six genes of close homology have been identified, with HYAL1 and HYAL2 being the main enzymes that contribute to the HA degradation [14]. Recently, a new enzyme with a similar activity has also been reported—TMEM2 [15]. HYALs have been long implicated in malignancies; however, their role can vary depending on the cancer type [16].

Over the past few years, attention has been paid on the involvement of proteolytic and glycolytic matrix enzymes in cancer progression. This article represents a thorough update of recent literature and focuses on the functional roles of major matrix remodeling enzymes in cancer initiation, propagation and progression as to highlight these key matrix partners as biomarkers for novel pharmacological targeting. Moreover, issues related with ongoing clinical trials are presented and critically discussed.

## 2. Matrix Metalloproteinases as Multitasking Players in Cancer Progression

ECM is a highly dynamic network that is present in all tissues and continuously undergoes controlled remodeling. This process is mediated by specific enzymes that are responsible for ECM degradation, such as MMPs [17,18]. MMPs proteolytically cleave a wide range of substrates, such as fibronectin, laminin, elastin and collagen, hence participating in normal processes including matrix turnover/homeostasis (during wound healing, angiogenesis and cellular migration), uterine involution, development, organogenesis, autocrine/paracrine signaling, apoptosis and autophagy. Moreover, they mediate the activation and deactivation of bioactive substrates, such as growth factor receptors (GFRs), cytokines and adhesion molecules that mediate inflammation, immunity and tissue repair. Because of their ability to degrade basement membranes and other ECM barriers, MMPs have been ascribed pathological roles in autoimmune disorders, cardiovascular disease, and carcinogenesis/cancer progression [19].

MMPs are modular enzymes consisting of a conserved catalytic domain and some ancillary domains responsible for enzyme localization and substrate selectivity. According to their structure and specificities, MMPs can be divided in five main subclasses: collagenases (MMP1, 8, and 13), gelatinases (MMP2 and 9), stromelysins (MMP3, 10 and 11), matrilysins (MMP7 and 26) and membrane-type MT-MMPs (MMP14, 15, 16 and 17). However, some new MMPs cannot be categorized in any of the above-mentioned groups [6]. They belong in metzincin clan where Zn^2+^ is responsible for the cleavage of peptide bond of the substrate [20]. MMPs are mainly secreted enzymes in the pericellular space and ECM, but some members are located on the cell surface [21]. They are synthesized as pre-pro-enzymes consisted of a signal peptide, a pro-domain, a catalytic domain, a linker and a C-terminal hemopexin domain responsible for substrate recognition. The latent zymogens are then proteolytically activated and interact with their endogenous tissue inhibitors (TIMPs) that regulate MMP enzymatic activity. MMPs may undergo posttranslational modifications such as glycosylation affecting their localization and substrate specificity [22].

Pericellular proteases mediate cancer progression through the communication of cancer cells with the surrounding cancer and normal cells including fibroblasts, macrophages, endothelial cells or pericytes etc. [9]. MMPs modulate the bioavailability of growth factors, such as epidermal growth factor (EGF) transforming growth factors (TGFα, TGFβ), fibroblast growth factor (FGF) and amphiregulin, and modulate the function of cell-surface receptors as E-cadherin and integrins. Moreover, they act to the direct cleavage of ligands for several growth factors, such as insulin-like growth factor (IGF) and the EGFR ligands that promote cancer cell proliferation [23]. In this way, MMPs orchestrate cell-cell and cell-matrix interactions thus being implicated in cancer progression as regulators of vital signaling cascades that control cancer cell properties including proliferation, motility, EMT, invasion and angiogenesis [24].

### 2.1. Matrix Invasion by MMPs

The metastatic niche of aggressive tumors starts with the degradation of the basement membrane and the subsequent invasion of cancer cells into the ECM and the vascular system. In order to degrade and remodel ECM, cancer cells hijack the components of normal cell invasion and develop dynamic protrusions of the plasma membrane, the so called invadopodia or invadosomes [25,26]. Key components for invadosome formation include three classes of proteolytic enzymes [Zn-regulated MMPs (MMP2, 9, 14 and ADAMs), cathepsin cysteine proteases and serine proteases], the actin regulators cortactin, Wiskott-Aldrich syndrome protein family members, the scaffold protein Tks5 and cofilin. Tks5 associates with numerous actin-remodeling proteins and proteases, mainly with ADAMs, to form invadopodia and promote tumor invasion in lung and breast cancer [27,28].

### 2.2. The Role of MT1-MMP in Breast Cancer Survival and Progression

**Figure 1:**
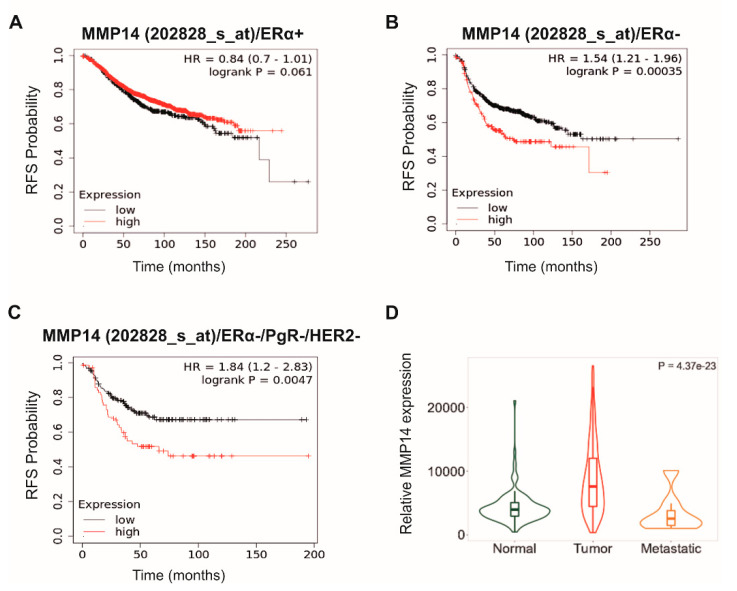
Prognostic and therapeutic value of MMP14 expression using meta-analysis tools. (**A**–**C**) Kaplan–Meier survival analysis of relapse-free survival (RFS) probability in different breast cancer patient datasets after diagnosis using gene chip data from GEO, EGA, TCGA databases. MMP14 (MT1-MMP) expression (**A**) is correlated to better prognosis in ERα-positive (ERα+) breast cancer patients following systemic treatment compared to ERα-negative (ERα-) patients (**B**) and triple-negative breast cancer patients (**C**). *p* value and hazard ratio (HR) value were calculated using a log-rank test [33]. (**D**) MMP14 gene expression profile of normal mammary tissues, breast invasive carcinoma and metastatic breast tissues, mining RNA-seq and gene chip data from GEO, GTEx, TCGA and TARGET databases, and presented as violin plot. Normal tissue N = 242, Tumor N = 7569, Metastatic N = 82. Abbreviations: EGA, European genome-phenome archive; GEO, gene expression omnibus; GTEx, genotype-tissue expression; TARGET, tumor alterations relevant for genomics-driven therapy; TCGA, the cancer genome atlas.

The transmembrane MT1-MMP (MMP14) has important roles in matrix turnover and regulates key functional properties of breast cancer cells, such as migration, invasion and angiogenesis. Moreover, MT1-MMP participates in MMP2 activation affecting breast cancer cell invasiveness [23]. Meta-analysis-based tools revealed that *MMP14* is closely related to the probability of survival in ΕRα-positive and ΕRα-negative breast cancer patients. Intriguingly, Kaplan–Meier survival analysis (online tool: http://kmplot.com, accessed on 2, February, 2021) revealed that the constitutive expression of *MMP14* in ΕRα-positive breast cancer patients following systemic treatment (Figure 1A) has the direction to a better probability of relapse-survival (RFS) compared to low *MMP14* expression. On the other hand, in ΕRα-negative breast cancer patients under systemic treatment (Figure 1B), *MMP14* expression related, in a statistically significant manner, to poor survival confirming its implication to the invasive and aggressive phenotype of ΕRα-negative breast cancer cells, such as in the case of triple-negative breast cancer (Figure 1C).

Differential gene expression analysis in tumor, normal and metastatic mammary tissues (TNM, https://www.tnmplot.com, accessed on 2 February 2021) revealed that in tissues from invasive breast carcinoma *MMP14* expression is remarkably higher compared to normal and metastatic tumors (Figure 1D).

Taking into consideration the above data, it is plausible to suggest the implication of MMP14 in the initiation of metastasis. This is proposed since this MMP is considered a master regulator of invadopodia functions and focal degradation of ECM, degrading fibrillar collagens and other matrix components on vesicular surfaces (laminin-5, integrins) and is the regulator of the first step of cancer cell invasion [29]. The dynamic nature of MT1-MMP and its effect on invadosomes is supported by its quick turnover in focal adhesions and may function to direct invadopodia assembly independently of its proteolytic activity in breast cancer cells promoting the metastatic potential [30,31,32].

### 2.3. Cancer Cell Aggressiveness and EMT

**Figure 2:**
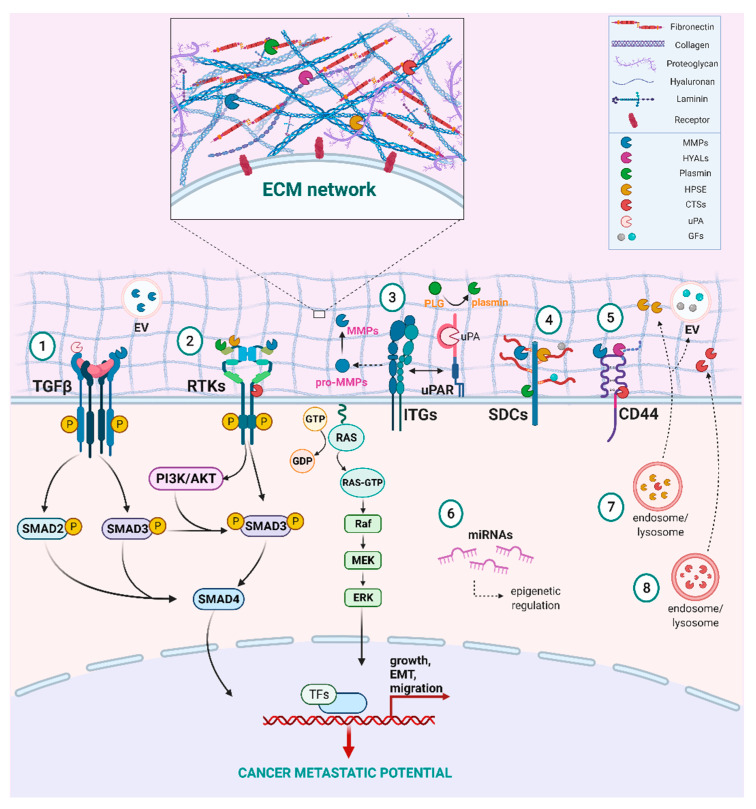
Main functions and signaling cascades mediated by matrix proteolytic enzymes. ➀ TGFβ induces the SMAD-dependent signaling to advance MMP expression. In turn, MMPs in the ECM, as well as uPA may promote the activation of TGFβ to induce cancer-associated EMT. ➁ Matrix remodeling enzymes interact with matrix effectors and affect RTKs activation, thus advancing downstream signaling cascades, like Ras-Raf-MAPK and PI3K/AKT, that lead to increased growth, migration and EMT. ➂ Integrins, major receptors responsible for cell adhesion, contribute to the conversion of pro-MMPs to their active forms. Besides, integrins also facilitate MMP synthesis and regulate their expression levels. Integrin signaling can also regulate the expression and localization of uPA and uPAR, while uPAR interacts with integrins and could act as co-receptor to induce migration and invasion. ➃ Plasmin accelerates syndecan (SDC) ectodomain shedding. ➄ HYALs degrade HA, altering its molecular size and initiating different biological responses, following HA binding to receptors such as CD44. ➅ miRNAs are critical regulators of matrix partners, either by inhibiting or promoting their expression. ➆ HPSE is activated in late endosomes and lysosomes by cathepsin L and stimulates the biogenesis and secretion of exosomes. Additionally, HPSE can be secreted in the ECM under the proper stimuli. ➇ Cathepsins reside in the lysosomes and upon rupture of the lysosomal membrane they are secreted in ECM to exert their actions. On the plasma membrane, they can shed the ectodomains of cell receptors and cleave growth factors and proteins (integrins, E-cadherin etc.). This figure was created using the tools available by BioRender.com, accessed on 2 February 2021.

The action of MMPs together with other major ECM enzymes in cancer progression is schematically represented in Figure 2. Gelatinases (MMP2 and 9) are known to be involved early in the tumor invasion degrading collagen in the basement membrane. MMP9 degrades type IV, V, XI and XVI collagen, gelatin, laminin, elastin, fibrillin and decorin [34], and also stimulates the release of growth factors including the TGFβ precursor and tumor necrosis factor alpha precursor (pro-TNFα) [35]. In this way, MMP9 is involved in photoaging and radial growth phase of melanoma development and tumor angiogenesis, and its overexpression has been shown to significantly contribute tumor invasiveness and spreading, being related with metastasis and poor prognosis [36].

MMP3 hydrolyzes various molecules such as type IV, V, IX and X collagen, proteoglycan, elastin, fibronectin, gelatin and laminins and can also trigger the activation of other MMPs like MMP1, 7, 9 and 13 promoting pulmonary adenocarcinoma, mammary ductal carcinoma and pancreatic cancer through Rac1b GTPase signaling axis [37]. Moreover, MMP3 has been highly expressed in melanoma and in ECM around blood vessels, proposing a significant role to remodeling induced by the aggressive melanoma progression [36].

MMP8 is mostly released from neutrophils and cleaves collagen type I and many other ECM components. MMP8 plays a crucial role in mediating response to various inflammatory conditions, cutaneous basal cell carcinoma development through TGFβ/SMAD2 axis [38]. Moreover, genetic studies on MMP8 have demonstrated that a single nucleotide polymorphism, like SNP rs11225395, was linked with a higher risk of melanoma growth [39]. In breast and oral tongue cancer, MMP8 inhibits cancer cell invasion and proliferation via cleavage of non-structural substrates. Conversely, in liver and ovarian cancers, high levels of MMP8 worsen the prognosis since it boosts TGFβ-induced EMT [40,41].

MMP9 is profoundly implicated in the invasion, metastasis, and angiogenesis of various tumors including non-small cell lung cancer, cervical cancer, ovarian cancer, and pancreatic cancer and can mediate the tumor microenvironment [42]. MMP9 promotes TGFβ1 induced EMT leading to increased migration and invasion capacity of thyroid [43], lung [44] and esophageal squamous [45] cancer cells. MT1-MMP promotes melanoma metastasis not only by ECM degradation, but also via regulation of genes involved in tumor cell progress and motility through the activation of MMP2/RAC1 signaling axis [46,47].

The overexpression of specific MMPs including MMP2, 3, 9, 13, 14 has been associated with the induction of EMT program and the acquisition of mesenchymal phenotype and increased aggressiveness of cancer cells. Overexpression of MMP1, 2, 3, 7, 9, 13, 14, 15 and 16 have been correlated to the invasive phenotype of melanoma cells [48].

TGFβ-mediated signaling axis is the principal mediator of EMT initiation and it has been recently revealed that TGFβ type I receptor activates Snail and MMP2 to promote tumor invasion [49]. MMP1, 2, 3, 7 and 9 proteolytically cleave the major adherens junction mediator, E-cadherin, promoting cell proliferation and differentiation of hepatocellular carcinoma [50,51]. In head and neck cancer the reduction of HA-CD44-mediated growth, migration and chemoresistance through Rho kinase-mediated signaling inhibits the activity of MMP2 and MMP9. Intriguingly, HA activates MMP2 and downstream Ras, Rho, PI3K, AKT signaling kinases promoting cancer invasion [52]. Moreover, MMP28 serves as a powerful EMT inducer by promoting the proteolytic activation of TGFβ and cell migration in lung carcinoma cells [53]. The role of ERβ in regulating the autocrine loop of aggressive breast cancer cells has been recently established. ERβ suppression inhibits EMT and reduces the expression levels of MMP1, 2, 7, 9 and 14 following reduced ERK1/2 activation stimulated by EGFR/IGF-IR and JAK2/STAT5-mediated migration in breast cancer cells [54].

### 2.4. MMPs Regulate Angiogenic Signals in Cancer Cells

Depending on substrate bioavailability and the time of expression, MMPs may regulate the angiogenic balance acting both as positive and negative regulators of angiogenesis by eliminating the physical barriers through ECM degradation. Degradation of VEGF by MMPs, specifically MMP1, 3, 7, 9, 16, 19 and MT1-MMP, regulates VEGF bioavailability and vascular patterning in tumors [55]. The proteolytic cleavage of insoluble VEGF by MMP9 enables interaction with its cell surface receptor triggering the angiogenic switch during carcinogenesis [56]. Interleukins (ILs) that regulate angiogenesis and metastasis are also processed by MMPs. For instance, MMP1, 2, 3 and 9 degrade the secreted IL-1β, whereas IL-8 secretion through EGFR and ERK1/2 activation requires MMP1, 8, 12, 13 and MT1-MMP activity [57]. Moreover, the degradation of collagen IV, XVIII and perlecan by MMP1, 2, 3, 9, 13 activates anti-angiogenic factors such as tumstatin, endostatin, angiostatin and endorepellin [58]. The types of MMPs, their functions and prognostic/therapeutic potential is summarized in Table 1.

### 2.5. Functional Relationship with GAG Chains

Intriguingly, there is a structure–function relationship between MMPs and GAGs that controls the specificity of proteinase/substrate interactions and stimulate GF signaling. MMPs direct interactions with the GAG chains of the PGs may function as allosteric regulators directing the release of GFs to the extracellular space promoting cancer cell proliferative properties [75]. In this context, it has been reported that the pro-MMP2 and MMP1 activation are enhanced by heparin [76].

The chondroitin-4-sulfate chains of melanoma-specific CSPG bind to the C terminus of pro-MMP2 to facilitate its activation by membrane-bound MMP16 [77]. Moreover, sulfated GAGs regulate MMP7 activation and its activity against specific substrates. MMP7 interacts with chondroitin-4,6-sulfate, HS and heparin chains on cell surface receptors [78] such as CD44 result in the proteolytic cleavage of heparin-binding epidermal growth factor precursor (pro-HB-EGF) and ErbB4 on the surface of lymphoma cancer cells [79]. The observation that the intracellular PG, serglycin that has established roles in EMT and oncogenic signaling [80], colocalizes in the same secretion granules as pro-MMP7, suggest that its CS chains may function as allosteric activators of pro-MMP7 [75].

### 2.6. Roles of Extracellular Vesicles EV-Associated MMPs during Tumor Progression

Extracellular vesicles (EVs) are secreted by most cells into the extracellular environment and have received the increased interest of nanomedicine and immunotherapy due to the delivery of their cargo [i.e., proteins, lipids, mRNAs, and microRNAs (miRNAs)] from origin cells to target cells. In this way, EVs serve as mediators of cancer cell properties [81]. EV-associated MMPs participate in ECM degradation directly and induce EMT, thus enhancing cancer cell migration, amoeboid migration and invadopodia maturation. Moreover, cancer cell- and stroma-derived EVs contribute to pre-metastatic niche formation through induction of ECM remodeling by travelling to distant sites [82,83].

In prostate cancer tissues, large oncosomes harbor abundant bioactive molecules involved in local invasion including MMP2 and 9 [84], while microvesicles shed by tumor cells are reported to deliver matrix ECM inducer (EMMPRIN) to fibroblasts, promoting tumor invasion and metastasis [85]. HSP90-loaded exosomes from metastatic breast cancer cells activate MMP2, leading to degradation of ECM molecules and release of growth factors, that boost signal transduction resulting in increased cancer cell invasion [86]. Moreover, stimulated endothelial cells also release EVs enriched in MMPs that initiate the proteolysis necessary for tumor cell invasion and angiogenesis [60].

MMP13-enriched exosomes derived from nasopharyngeal cancer cells promote cell motility and invasion with increased expression of major EMT markers (E- and N-cadherin) [64]. Global proteomic profiling of exosomes from MDCK and 21D1 cells has revealed that mesenchymal 21D1 exosomes are enriched in metalloproteinases including MMP1, MT1-MMP and MMP19, ADAM10 and ADAMTS1 and α3, α6 and β1 integrins [87].

Several metalloproteinases such as MMP2, MMP3, MMP9 and MT1-MMP have been shown to be upregulated in lymph nodes or lung tissues distant from the primary tumor by tumor-derived exosomes [88]. Specifically, MT1-MMP in EVs is crucial to increased cell motility and ECM degradation [89]. Intriguingly, highly abundant exosomal miRNAs such as miR-100-5p, miR-21-5p, and miR-139-5p from prostate cancer stem cells increased MMP2, 9, 13 and RANKL expression and enhanced the migration of fibroblasts, contributing to local invasion and pre-metastatic niche formation [90].

### 2.7. MMP-Mediated miRNA Alterations in Tumor Progression

Membrane vesicles that carry and transfer secreted miRNAs, control cell-cell communication and subsequent signaling pathways in recipient cells, underlying the significance of miRNA involvement in tumor progression [91]. It is well established that miRNA regulate the expression of major ECM components via targeting conserved seed sequences in the 3′ UTRs of target mRNAs, serving as biomarkers and targets for several cancer types, since they may act as oncogenes or as tumor suppressors depending on the biological functions of their target mRNAs [92,93,94].

Major ECM molecules regulate the expression and the subsequent functions of specific miRNAs. Asuthkar et al. reported that MMP9 drives miR-494 suppression resulting in augmented syndecan-1 shedding and increased angiogenesis in medulloblastoma cells [95]. Recent data suggest that estrogen receptors (ERs) are critical mediators of miRNAs that modulate matrix composition and signaling [96]. The ERα/β-mediated miR-10b and miR-145 increased MMP2, 7 and 9 expression following overexpression and inhibition, respectively, in ERβ-suppressed MDA-MB-231 breast cancer cells which resulted in increased ERK1/2 activation and aggressive phenotype [97]. Overexpressing the ER-mediated miR-200b resulted in reduced expression and activity of MMP2, 9, 7 and 14 and ERK1/2 phosphorylation, while having no significant effects in ERα-positive MCF7 breast cancer cells [98]. Collagen type I-mediated let-7 in pancreatic ductal adenocarcinoma involves TGFβ1-mediated MT1-MMP expression which drives fibrosis and promotes pancreatic cancer [99]. The downregulation of miR-21 in glioma cells leads to decreased migratory and invasive capacity by targeting MMP inhibitors, such as TIMP3 [100]. Exosomal miR-21 upregulates MMP2, 9 and 11 and increases proliferation, invasion and chemoresistance of colon adenocarcinoma cells [101]. Another study reported that exogenous expression of miR-29b regulates prostate cancer cell growth by modulating antiapoptotic and prometastatic ECM molecules, specifically by directly targeting and suppressing MMP2 in prostate cancer cells [102].

Elevated levels of miR-145-5p resulted in decreased epithelial ovarian and cancer cell migration through MMP2 and MMP9 downregulation [103,104,105]. Moreover, MT1-MMP expression in pancreatic cancer cells has been correlated to the loss of miR-200a, b and c, which leads to increased cell growth, invasion, migration and aggressiveness of cancer cells [106]. MiR-155 promotes breast cancer cell proliferation and migration via MMP16 targeting [107]. Last but not least, miR-206 downregulates MMP2 and MMP9 leading to suppressed migration and invasion in MDA-MB-231 breast cancer cells [108].

### 2.8. Pharmacological Targeting of MMPs

Concerning the diversified, functional, yet vital roles of MMPs in tumor progression, invasion and angiogenesis, MMPs are highly important when considered as drug targets in cancer. A growing body of evidence is advocating for the complex signaling pathways that MMPs mediate; thus, the establishment of new strategies to design novel next generation MMP inhibitors (MMPIs) is needed to topically inhibit a single MMP in a delicate way with high selectivity and specificity. Several generations of synthetic MMPIs have been tested in phase III clinical trials including peptidomimetics, non-peptidomimetics, tetracycline derivatives, natural compounds, monoclonal antibodies (mAbs) and antisense strategies [6].

The first generation of MMPIs were designed to target the active Zn site of MMPs that is the most common way to inhibit these enzymes. Several MMPIs were developed and tested in several cancer types, including phosphinates, hydroxamates (i.e., batimastat, marimastat), aminocarboxylates, carboxylates and sulfhydryl groups. Chemically modified tetracyclines include metastat, minocycline and doxycycline. Periostat, a broad spectrum MMPI with higher specificity at MMP1, 2, 8 and 9, has entered phase II trials for resectable pancreatic cancer [109]. However, early MMPI strategies (i.e., peptidomimetic and non-peptidomimetic analogs) failed their way to clinical trials since the metal chelating group resulted in unspecific inhibition, as the novel compounds targeted common features in MMPs, and low substrate selectivity in vivo [110]. SB-3CT small-molecule inhibitor that reforms the proenzyme structures of MMP2 and 9 by binding the enzyme catalytic sites, is reported to exhibit antiangiogenic and antimetastatic properties [111], while enhancing the efficacy of immunotherapy [112]. NSC405020 is a novel small-molecule MT1-MMP inhibitor repressing its pro-tumorigenic activity in vivo [113]. Other MMPIs focusing on malignancies and tested in clinical trials include neovastat, which strongly inhibits MMP2 activity (metastatic kidney cancer, advanced colorectal/breast cancer) [114] and BMS-275291, a wide-spectrum MMPI (lung, breast cancer) [109,115].

Since several broad-spectrum MMPIs have failed in the clinical trials, an alternative approach involves the design of macromolecule inhibitors targeting domains beyond the conserved catalytic cleft (exosites) of the enzymes. This led to the development of antibody-based MMPIs that interact not only with the catalytic domain, but also with surface regulatory hotspots, rendering a highly specific inhibition while maintaining the activity of other MMPs [116]. A set of mAbs against MT1-MMP have been developed, such as LEM-2/15, -2/63, -1/58, inhibiting MT1-MMP catalytic activity in a highly specific manner [117]. The development of the mAb LEM-2/15, which targets the exposed loops of the catalytic domain of MT1-MMP with high affinity overcoming cross-reactivity with other MMP members, impairs ECM protein degradation and is a promising strategy to control MT1-MMP activity at the leading edge of migratory cancer cells since it has anti-angiogenic properties and reduced tumor cell invasion [118]. Therapeutic promise has been shown with the full-length mAb, REGA-3G12, which targets the catalytic cleft of MMP9 while the MMP2 activity remains unaffected [119]. The selective MMP2/9 inhibitory antibodies, SDS3 and SDS4, have been designed to mimic the exposed catalytic machinery in the active form of MMPs but had no inhibitory activity toward MMP1, 7 and 12. SD3 demonstrated therapeutic potential in mouse colitis models [120].

ECM homeostasis is an extremely delicate balance and has to be maintained in order to avoid leading to pathological situations caused by unwanted complications. For instance, it has been demonstrated that even the most selective MMPIs that inhibit pathological signaling cascades, disrupt tissue homeostasis and influence their targets both in healthy and in tumor tissues in the case of cancer. On top of that and in order to improve the extremely challenging MMP inhibition in vivo, novel approaches have been designed exploiting the benefits of protein engineering and nanotechnology. Fab fragments of antibody against the MT1-MMP were modified at distal end of polyethylene glycol (PEG) of doxorubicin-encapsulating liposomes suppress tumor growth in vivo [121]. Recently, a functionalized liposomal nanocarrier containing a mAb with a MMP2 cleavable peptide that recognizes the cancer cells has been designed, which releases the encapsulated therapeutic compound that has cytotoxic effects for cancer cells [122]. An interesting example of nanocarriers, includes nano-heparin analogue from *Styela plicata*, that inhibits cell proliferation, migration, and invasion and induced apoptosis in breast cancer cells and this was followed by reduced MT1-MMP and uPA expression [123]. Multifunctional mesoporous SiO_2_ nanoparticles were loaded with β-cyclodextrin and an MMP substrate peptide in order to selectively target the MMP-rich hepatocellular carcinoma cells and subsequently induce the intracellular release of the cytotoxic drug [124].

## 3. Plasminogen Activation System in Cancer Metastasis

PLG, a 92 kDa single-chain glycoprotein, is mainly produced in the liver and contains glutamic acid as the N-terminal amino acid (Glu-plasminogen). Its structure is comprised by seven distinct domains; a serine protease domain, an N-terminal activation peptide and five kringle domains, triple-looped structures of approximately 80 residues with three disulfide bonds, which mediate the binding with cell surfaces, extracellular ligands and cell receptors and confer high binding specificity of plasminogen to fibrin. [125]. PLG’s conversion to plasmin results in a broad-spectrum serine protease that can degrade not only substrates like fibrin but also ECM proteins, including fibronectin and laminin through MMPs activation [126]. In turn, plasmin can be inhibited by α2-antiplasmin and α2-macroglobulin [127]. In humans, two main plasminogen activators, members of the serine protease family, have been identified, urokinase plasminogen activator (uPA) and tissue-type plasminogen activator (tPA).

tPA is a *ca* 70 kDa glycoprotein, synthesized by vascular endothelial cells of different tissues and comprised of five domains, with 17 disulfide bridges to maintain its conformation [128,129]. The N-terminal region includes a finger domain responsible mainly for the binding of tPA to fibrin, but also allows interactions with membrane receptors like low density lipoprotein receptor-related protein (LRP) and annexin II [130,131]. The four other domains are an EGF-like domain, two kringle domains, that encompass an active site with high affinity for lysine, and the catalytic domain of the protease responsible for plasminogen activation to plasmin [132].

uPA has a molecular weight of approximately 50 kDa and is synthesized in cells as pro-uPA. Pro-uPA contains a single-chain non-active glycoprotein with three domains, an EGF-like domain responsible for the receptor binding, a kringle domain, and a catalytic serine protease domain at the C-terminal region. Upon secretion, it is cleaved to form the active high molecular weight uPA (HMW-uPA), which can be further cleaved into low-molecular weight uPA (LMW-uPA). Binding of uPA to cell surfaces is usually mediated by the uPA receptor, uPAR, through its growth factor domain [133]. uPAR is a heavily glycosylated molecule and its sequence contains various glycosylation sites [134]. The main endogenous inhibitor of uPA and tPA, PAI-1, belongs to the serpine protease inhibitors (serpins) superfamily and is produced in a variety of cell types and distributed in many tissues [135]. PAI-1 is usually present in its latent, inactive conformation which is activated upon binding with vitronectin [136].

The PA system is implicated in various physiological processes, including tissue regeneration, wound healing, and clot dissemination; hence, any deregulation can potentially lead to pathological implications. One of the most well-studied diseases caused by plasminogen deficiency is ligneous conjunctivitis, a disease which causes characteristic fibrinous, thick, woody deposits on mucosal surfaces [137].

### 3.1. The PA System in Cancer

In cancer, the PA system has been widely linked with tumor initiation and progression (Table 2). Proteomics analysis of the HCT116 cell line, known for *KRAS* and *PI3KCA* mutations common in colorectal cancer, showed that altered expression of uPAR is associated with modifications in major hallmarks of cancer including resisting cell death, invasion/metastasis and sustaining proliferation, as it affects the ubiquitin-proteasome system and the major cancer-related pathways ERK/MAPK, STAT3, PTEN as well as the Rho GTPases-mediated signaling (Figure 2) [138]. Hau et al. showed that in urinary bladder carcinoma uPAR is overexpressed in higher grade invasive tumors. Additionally, uPAR gene-silencing in invasive human bladder cancer cell lines downregulated cell migration and invasion via regulation of mTORC2 activation [139]. Immunohistochemical analysis of tissue specimens from patients with prostate cancer that underwent radical prostatectomy revealed significant overexpression of uPA, uPAR and PAI-1 that was linked with biochemical recurrence [140].

Furthermore, the uPA/uPAR axis associated with CAFs contributes to cell invasion, aggressive behavior and poor outcome in multiple myeloma. In a recent study, Kubala et al. explored the connection between PAI-1 mediated inflammation and pro-tumorigenic activity within the tumor microenvironment (TME). The authors showed that PAI-1 promotes macrophage migration through its LRP1 interacting domain, while it polarizes macrophages toward an M2 anti-inflammatory phenotype through uPA interacting domain. Hence, the dual function of PAI-1 on monocytes/macrophages is supported by two distinct domains, one affecting migration and the other polarization. In addition, PAI-1 also activates a p38MAPK/NF-κB/IL-6 loop in a paracrine manner to promote M2 macrophage polarization. Since IL-6 is known to increase PAI-1 expression [141], activation of p38MAPK/NF-κB/IL-6 by PAI-1 could lead to a positive feedback loop in which increased IL-6 secretion results in more PAI-1 production [142]. Additionally, Bydoun et al. determined that, upon EMT induction by TGFβ1, PAI-1 was significantly upregulated in A549 non-small cell lung carcinoma (NSCLC) cells and this effect was modulated via FOXC2-mediated PI3K signaling as well as SMAD4-dependent TGFβ1 signaling [143].

S100A10, a protein that acts as plasminogen receptor, has gained attention in recent years and studies has shown its involvement in the induction of invasion and metastasis. Specifically, S100A10 in a heterotetramer with Annexin II can act as co-receptor for tPA, plasminogen and pro-cathepsin B to promote invasion and metastasis in breast carcinoma and glioma cells, but it can also affect macrophage invasion and breast cancer cell proliferation [144]. In addition, expression of S100A10 in macrophages is crucial for their employment in primary tumor formation sites in fibrosarcoma and lung carcinoma models [145]. Likewise, key molecules of the PA system interact with other degrading enzymes to advance cancer progression. Studies have concluded that plasmin modulates angiogenesis via activation of MMPs as shown in in vitro and in vivo models (Figure 2). Additionally, the crosstalk between uPAR, TGFβ and MMPs is implicated in cancer related EMT and activation of pro-tumorigenic signaling pathways in advanced cancer [146] (Figure 2).

### 3.2. Pharmacological Targeting of Plasminogen Activation System in Cancer

Various therapeutic interventions that target the PA system have been tested through the years, though most of them concerning other pathologies, like cardiovascular diseases and not cancer. Targeting uPA-uPAR interactions is usually achieved by small molecules or peptides derived from the uPAR-binding region of uPA. Å6, an example of uPA-derived peptide, contributed in reduced angiogenesis, growth and metastasis in different experimental models of breast and prostate cancer, as well as in preclinical studies [147,148,149]. Besides, it has also been tested in phase I and II clinical trials for ovarian cancer treatment [150].

Likewise, animal models treated with anti-uPAR antibodies, designed to block uPAR-ligands interactions, displayed reduced growth rates and invasion [151]. Recently, upamostat, a uPA inhibitor, was approved by FDA for treatment in pancreatic cancer, after enhancing the survival rates of the patients in phase II clinical trials [152]. Given the wide involvement of the PA system in tumor initiation and progression, further research is needed that will utilize newly synthesized inhibitors or already approved pharmaceuticals against the PA system in cancer.

## 4. Cathepsins in Tumor Progression

The cathepsin family of lysosomal proteases is a super-family of proteolytic enzymes with 15 members to date. Cathepsins are usually classified into cysteine (B, C, F, H, K, L, O, S, V, Z/X and W), serine (A and G) and aspartic (D and E) proteases, depending on the catalytic site residue and can be further subdivided in endo-peptidases (S, K, V, F, L) and both endo and exo-peptidases (B, C, H, Z/X), based on their proteolytic activity [173,174].

Cathepsins are first synthesized in the rough endoplasmatic reticulum (ER) as pre-proenzymes and converted to inactive zymogens upon cleavage of the N-terminal signal Golmehr when entering the ER lumen [175]. Cathepsins are then transferred to late endosomes/lysosomes where the acidic environment promotes propeptide cleavage and full activation of the mature enzymes [159]. Notably, the propeptide acts as a reversible inhibitor, even in its cleaved, soluble form, hence representing a critical checkpoint of cathepsin activation [176]. The highly conserved mature domain is a catalytically active region comprised of 214–260 amino acids and encompasses the active site that includes the very common triad of cysteine, histidine and asparagine residues (CHN motif) [159]. The activity of cathepsins is usually under strict control by their naturally occurring endogenous inhibitors, cystatins. Cystatins are divided in three different types (stefins, cystatins, kininogens) with similar structures and modes of action [177].

### 4.1. The Role of Cathepsins in Cancer Progression

Under physiological conditions, cathepsins are mainly localized in lysosomes, a compartment with acidic pH, optimal for their activation, where they play role in immune response, antigen processing, autophagy and intracellular protein degradation. Rupture of the lysosomal membrane, induced by reactive oxygen species (ROS) or other stimuli, allows cathepsins to translocate to other cellular compartments and exert their actions, even though the pH in these compartments is neutral and, as such, not optimal [178]. In tumors, cathepsins are often found overexpressed and have been recognized as predictive markers for disease progression and therapy response [168,179]. Commonly, this overexpression of cathepsins leads to their secretion from cancer cells and tumor-associated macrophages (TAMs) in TME and subsequent aberrant ECM remodeling. The main functions of cathepsins in tumors are summarized in Table 2.

Secreted cathepsins advance ECM remodeling through degradation of key matrix proteins, such as fibronectin, laminin, collagen and aggrecan. Recently they have also been found to have sheddase activity (Figure 2). Specifically, cathepsins B, S and L target E-cadherin, a pivotal molecule in cell-cell adhesion, hence advancing tumor invasion [159]. Similarly, cathepsins L and S shed perlecan, a proteoglycan that promotes angiogenesis. In MDA-MB-231 breast cancer cells, CD44, neuropilin 1, plexin A1 and B2, EGFR, as well as various CAM proteins, namely nectin-like protein 5, ALCAM, L1CAM and MUC18, were identified as cathepsin substrates [180].

In breast invasive ductal carcinoma (IDC) patients, analysis of tissue specimens by IHC revealed that CTSL is expressed in advanced tumor stage patients, CTSB is found in ERα+ tissues with poor disease-free survival (DFS) rates, CTSK is expressed in ERα+/PR+ tumors and, finally, CTSD expression associates with poor prognosis, high incidence of metastasis, especially to the chest, and low DFS rates [181]. Likewise, CTSV expression favors distant metastasis, hence low survival rates in ERα+ breast cancer, as it facilitates cell proliferation and invasion while attenuating the expression of GATA3 through PI3K-AKT-GSK3β pathway [170] (Figure 2). In addition, in MCF-7 breast cancer cells CTSB is upregulated while it suppresses E-cadherin. Overexpression of CTSZ/X in hepatocellular cancer enhances motility and invasion via inhibition of E-cadherin and induction of fibronectin, while secretion of CTSB and CTSL was promoted by Abl/Arg nonreceptor tyrosine kinases in melanoma cell lines [182]. Release of CTSB is also important for increased invasion and cancer progression in pancreatic ductal adenocarcinoma, glioma and esophageal adenocarcinoma. Proteomic analysis and western blot assay showed that CTSB is highly overexpressed in papillary thyroid cancer (PTC) cells compared to epithelial thyroid cells and regulates EMT and metastasis via p38 activation. In addition, in clinical samples obtained by PTC patients, CTSB expression was correlated to lymph node metastasis [183]. Gut microbiota imbalance in colorectal cancer, stimulates CTSK secretion which, in turn, mediates the TLR4-dependent M2 polarization of TAMs to promote tumor metastasis in a positive feedback loop [164]. Moreover, upregulation of CTSL in A549 human lung carcinoma cells induces the expression of EMT-associated transcription factors and mediates cisplatin and paclitaxel chemoresistance [167]. Furthermore, CTSK has been found poorly expressed in normal prostate and in the prostate epithelial cells, whereas the expression was higher in the non-metastatic LNCaP cells and even greater in C4-2B and PC3 cells, and in skeletal metastatic tumor as revealed by tissue microarray. To this extent, CTSK affirmed as a key factor specific in PCa skeletal metastasis and PCa-induced bone lesions in vivo [165]. In a study by Yu et al, the role of cathepsins in a multistage epithelial carcinogenesis was examined using K14-HPV16 transgenic mice. During the evolution of premalignant dysplasia, they noticed elevated expression of CTSS, but reduced expression of the cathepsin endogenous inhibitor cystatin C in the skin tissue extract. The authors concluded that higher cathepsin expression and activity in cystatin C-deficient mice may contribute to the evolution of dysplasia by changing premalignant tissue epithelial proliferation, apoptosis, and neovascularization [184]. More recently, in a study by Wäster et al., the impact of ultraviolet radiation for increased melanoma dissemination was investigated. The authors showed that, besides the degradation of ECM, an additional role of cathepsins in the melanoma cells may be the activation of TFGβ1, which subsequently promotes the upregulation of fibroblast activation protein-alpha (FAP-α)-mediated cancer cell spreading [185].

### 4.2. Pharmacological Targeting of Cathepsins

Recently, cathepsin-related research has been widely focused on small molecules and peptides as therapeutic options. Anantaraju et al. combined molecular docking and in vitro studies and were able to single out 17 small non-peptidic molecules which, via inhibition of CTSD, downregulated growth rates in MCF-7, MDA-MB-231, MDA-MB-468, and SK-BR-3 breast cancer cells [186]. Saito et al. developed the p14 MIS peptide, a combination of the p14^ARF^ mitochondrial targeting protein and CTSB proteolytic cleavage site, and used it as treatment in vitro in pancreas adenocarcinoma, uterine squamous cell carcinoma, lung adenocarcinoma, breast adenocarcinoma, colon adenocarcinoma, and hepatocellular carcinoma cell lines, but also in vivo, in a xenograft mouse model. Their results revealed suppression of in vitro growth rates and in vivo tumor growth and invasion in normal tissues [187]. Furthermore, carrier-free nanoparticles of cathepsin B-cleavable peptide (Phe-Arg-Arg-Gly; FRRG)-conjugated doxorubicin (DOX) prodrug (FRRG-DOX) were tested in CTSB-overexpressing human colon adenocarcinoma (HT-29)-bearing mice and the data showed great therapeutic efficacy of the FRRG-DOX nanoparticles with low toxicity, hence such carrier-free nanoparticles could be potentially used for targeted clinical applications [188]. Whilst there is a slew of preclinical trials describing anticancer effects of numerous inhibitors in animal models, the complexity of cathepsin functions combined with the fact that they are mostly produced by TAMs results in limited clinical evaluation of cathepsin inhibitors in cancer. Currently, only odanacatib, a CTSK inhibitor, has been evaluated for breast and prostate bone metastasis but the clinical trials were discontinued [189]. Hence, it is becoming evident that more research is needed towards the development of new therapeutics that will target cathepsins.

## 5. Non-Proteolytic Enzymes in ECM Remodeling

### 5.1. Heparanase

HPSE is an endo-β-D-glucuronidase that cleaves the HS side chains of HSPGs [190]. Initially, HPSE is translated as an inactive pre-pro-enzyme and, following removal of the signal sequence, the latent pro-HPSE is proteolytically modified into the active enzyme heterodimer by cathepsin L [191,192]. Active HPSE selectively degrades the linkage between the D-glucuronic acid and the N-sulfo-D-glucosamine residue sulfated at C-3 and C-6, producing fragments of 5–10 kDa [193]. Under physiological conditions HPSE resides in late endosomes and lysosomes; however, in response to proper stimuli it can be secreted in the ECM via activation of protein kinase A and C [194]. Once there, HPSE can participate in ECM degradation and remodeling affecting the ECM structures and/or through the release of HS-bound ligands such as growth factors and cytokines. Notably, HPSEcan exert non-enzymatic action by activating signaling cascades. A close homolog of HPSE, HPSE-2, also exists but lacks enzymatic activity [195]. The modes of HPSE action are schematically represented in Figure 2.

#### 5.1.1. Involvement of Heparanase in Cancer Cell Properties and Tumor Metastasis

**Figure 3:**
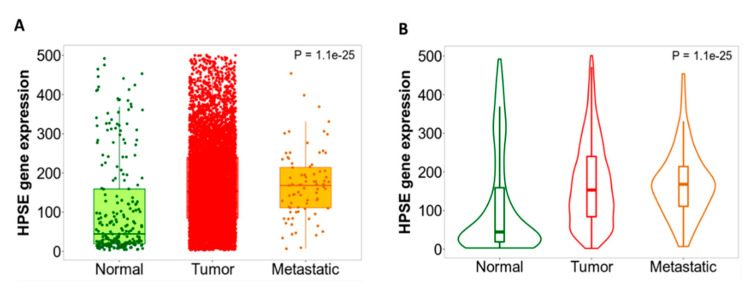
HPSE gene expression profile of normal mammary tissues, breast invasive carcinoma and metastatic breast tissues mining gene chip data from Gene Expression Omnibus (GEO) repository and presented as boxplot (**A**) and violin plot (**B**). Normal tissue N = 242, Tumor N = 7569, Metastatic N = 82 [59].

HPSE is expressed in low levels in normal cells, with its presence restricted to platelets, mast cells, placental trophoblasts, keratinocytes and leukocytes [13]. However, its expression is elevated in a multitude of malignancies and usually correlates with disease aggressiveness and increased metastatic potential (Figure 2). A differential gene expression analysis performed in normal, tumor and metastatic mammary tissues using the TNMplot web tool (https://www.tnmplot.com, accessed on 2 February 2021) reveals significantly higher HPSE levels in malignancy compared to normal tissues (Figure 3A,B). Additionally, high HPSE levels are often a signal for poor prognosis in several cancers including breast, ovarian, pancreatic, gastric, lung and melanoma [194,196,197,198,199,200].

The role of HPSE in cancer is mainly attributed to its HS degrading activity, which facilitates cell invasion and propagates metastasis. In gastric cancer, HPSE aids in the degradation of basement membrane, which allows tumor invasion and metastasis [201]. It is noticing that HPSE may impact the aggressive tumor phenotype due to its effect on increasing the expression and shedding of the HSPG syndecan-1. HPSE cleaves the HS side chains of syndecan-1, leaving the core protein vulnerable to proteases. In myeloma cells elevation of HPSE expression upregulates MMP9 expression via ERK phosphorylation. In addition, uPA and uPAR expression levels are also increased, suggesting a significant role for HPSE in syndecan-1 shedding. HPSE also plays an important role in promoting angiogenesis, primarily via the release of HS-bound growth factors such as VEGF and FGF [202]. Moreover, HPSE has been connected to inflammation through the release of pro-inflammatory cytokines. Since inflammatory conditions favor tumor initiation, it is proposed that HPSE is a possible mediator in inflammation-driven cancer. Specifically, HPSE sustains the activation of macrophages that supply cancer-promoting cytokines such as TNFα, IL-1 and IL-6, thus shaping the tumorigenic microenvironment [203]. Another important process implicated in tumor metastasis is the induction of EMT. HPSE has been reported to enhance the expression of the EMT markers vimentin, fibronectin and RANK in multiple myeloma cells and consequently their motility, partly due to activation of the ERK signaling pathway [204]. The main functions of HPSE in cancer progression are summarized in Table 3.

#### 5.1.2. Heparanase Regulation of Exosome Formation and Autophagy in Cancer Progression

In many cancer cases, both HPSE and exosome release are elevated, often correlating with aggressive tumor phenotypes [218]. Indeed, upregulated levels of HPSE stimulate the secretion of exosomes in a concentration-dependent manner, while also altering their content and functionality. Specifically, it is the enzymatic activity of HPSE driving the effect, as exosome biogenesis is regulated by the syndecan-syntenin-ALIX pathway and HPSE is implicated in its activation [219]. Moreover, HPSE-overexpressing cells have their exosome cargo impacted as they include higher levels of syndecan-1, VEGF and HGF compared to their low HPSE-expressing counterparts. This change in composition likely reflects the change in cell behavior as well since these exosomes stimulate spreading and invasion of both tumor and host cells, driving them toward behaviors associated with enhanced tumor survival and progression [206]. Evidence also supports the role of HPSE in chemoresistance and eventual patient relapse. Exosomes secreted during anti-myeloma therapy contain a high level of HPSE that degrades ECM and interacts with neighboring tumor or host cells [207]. Additionally, the delivery of HPSE through exosomes may also impact distal sites by establishing a pre-metastatic niche and thus supporting metastasis.

Another mechanism by which HPSE can enhance tumor progression and chemoresistance, at least in part, is by promoting autophagy [220]. HPSE, which normally resides within lysosomes, can be colocalized with endogenous LC3-II, a protein involved in the formation of autophagosomes. Following HPSE overexpression in tumor-derived cells increase in autophagy is observed, while also rendering them more resistant to stress cues and chemotherapy [221]. While autophagy is a process required for maintaining cellular homeostasis, it confers growth advantages to cancer cells under stress, thereby exerting pro-tumorigenic action. Even though the mechanism governing this HPSE-driven autophagy is not yet elucidated, it most likely involves a reduction in mTOR1 activity.

#### 5.1.3. Roles of miRNAs in Heparanase Expression

During tumor progression, HPSE expression can also be regulated by miRNAs. The expression of miR-1258 has been inversely correlated with HPSE levels in breast cancer and non-small cell lung cancer, with its overexpression resulting in reduced brain metastasis and invasiveness, respectively [222,223]. Moreover, miR-429 exhibits a reduced expression in gastric cancer tissues and its overexpression inhibits the transcription and translation of the *HPSE* gene, decreasing their invasive properties [224]. In both cases, the miRNAs bind to the 3′ UTR of HPSE, altering its expression directly. On the other hand, high miR-558 levels induce HPSE expression in gastric cancer cells and neuroblastoma by targeting the *HPSE* promoter and thus facilitating tumor progression [225,226].

#### 5.1.4. Heparanase Inhibition as an Anti-Cancer Strategy

Considering the upregulation of HPSE in most cancers, its unique role in HS degradation and involvement in tumor progression, it is sensible to view it as an attractive target for the development of cancer therapies. Naturally, HPSE can be inhibited by the HS-resembling heparin; however, the latter’s potent anticoagulant activity makes it unsuitable for an anti-cancer drug. Therefore, current HPSE inhibitors revolve around chemically modified HS mimetics with non-anticoagulant effects. Several preclinical models have promoted the anti-cancer potential of HPSE inhibition and clinical trials have been carried out for the HPSE inhibitors PI-88 (muparfostat), PG545 (pixatimod), SST0001 (roneparstat) and M-402 (necuparanib) [227].

PI-88 (muparfostat) exhibits anti-angiogenic and anti-metastatic effects by inhibiting the HPSE enzymatic activity and blocking FGF1, FGF2 and VEGF interactions. It has been the subject of several phase I and II clinical trials as a single agent or in combinational therapies and even reached phase III for the treatment of hepatocellular carcinoma, showing promising signs but ultimately unsuccessful results in disease-free survival of the participants [228,229,230]. Another HS mimetic, PG545 (pixatimod), stimulates innate immune anti-tumor responses in a variety of preclinical cancer models. A phase I trial for patients with advanced solid malignancies demonstrated partial disease control, paving the way for use in combination with existing therapies [231]. Anti-myeloma chemotherapy has been linked with upregulation of HPSE expression, leading to enhanced tumor growth and chemoresistance. SST0001 (roneparstat) diminishes these effects in hematological malignancy models; however, a phase I clinical trial revealed little efficacy in multiple myeloma treatment [232,233]. Finally, the heparinase inhibitor M402 (necuparanib) attenuates metastasis and prolongs survival in murine cancer models but phase I and II trials in combinational anti-cancer therapy of metastatic pancreas cancers yielded no significant effects in overall survival [234,235].

Beyond the HS mimetics, the nonsteroidal anti-inflammatory drug aspirin, widely suggested to have an anti-cancer effect over long-term use, directly binds to the HPSE active site inhibiting its enzymatic activity and impedes HPSE-dependent cancer cell migration, metastasis and angiogenesis both in vitro and in vivo [236]. As attractive HPSE inhibition may seem for cancer attenuation, it is of importance to note the critical role of the enzyme on the infiltration of activated NK cells to primary tumors and metastasis sites [237]. Consequently, potential inhibitors must be highly selective and extensively researched to limit adverse effects.

### 5.2. Hyaluronidases

HYALs are endo-β-N-acetylglucosaminidases that degrade HA via hydrolysis of the β(1,4)-glycosidic bond between the D-glucuronic acid and N-acetyl-D-glucosamine [238]. The family of HYALs traditionally contains six extensively homologous genes: *HYAL1-4*, *HYALP1* and *SPAM1*. HYAL1 and HYAL2 constitute the predominant isoforms to cleave HA and their activity is favored in acidic conditions. HYAL1 is found in lysosomes but can also be secreted in the ECM and degrades HA in small fragments of *ca* 800Da. HYAL2 is a GPI-anchored protein of the cell membrane that that produces HA fragments of *ca* 20kDa. Interestingly, HYAL2 colocalizes with the main HA receptor CD44 and the produced oligosaccharides can be internalized to be further degraded by HYAL1 [239,240]. The product of *SPAM1* is PH-20, a HYAL located on the sperm acrosome which facilitates fertilization, whereas *HYALP1* is a pseudogene that is not translated. Finally, HYAL4 cleaves chondroitin sulfate while HYAL3 shows no activity towards HA [241,242]. Recent studies also point to transmembrane protein 2 (TMEM2) as a potent hyaluronidase of the cell surface, which degrades HA into fragments of *ca* 5kDa and functions at a pH of 6–7 [243]. In addition, the cell migration-inducing and HA-binding protein (CEMIP), also known as KIAA1199 or HYBID (HA-binding protein involved in HA depolymerization) exhibits a HA-depolymerization activity which depends on the clathrin-coated pit pathway [244]. While HYALs often correlate with cancer progression, they have also been shown to have tumor suppressive effects. Their mode of action is closely related to the degradation of HA, since HA related effects depend on its molecular size [245].

#### 5.2.1. The Role of Hyaluronidases in Cancer Progression

Depending on its molecular size, HA exhibits different biological properties and functions [16,245]. Consequently, HA degradation by HYALs produces fragments that control a variety of processes (Figure 2). In particular, low molecular weight HA (LMW-HA) accumulation is associated with tumor aggressiveness and angiogenesis [246]. Increased HYAL1 expression correlates with bladder cancer diagnosis and promotes tumor invasion and metastasis, constituting a potential prognostic indicator for progression and recurrence [247,248]. Moreover, *HYAL1* is a direct target of ERα in breast cancer cells and it is repressed by estrogen [208]. In invasive melanomas the tumor cells exhibit markedly reduced amount of HA attributed to the increased expression of HYAL2 [249]. Knockdown of HYAL1 in pancreatic cancer cells significantly decreases pancreatic cancer cell migration in the presence of high molecular weight HA (HMW-HA), indicating a HYAL1-dependent mechanism by which the newly formed LMW-HA derivatives promote motility [250]. Another aspect of HYAL involvement in cancer progression is through the means of extracellular vesicles. Active HYAL1 is present in exosomes of prostate cancer cells and enhances cell motility by engaging FAK-mediated integrin signaling, possibly priming the nearby stromal cells into a pro-invasive phenotype [211].

Contrary to the tumor-progressing narrative of HYALs, a suppressive role for HYALs in metastasis has also been shown. HYAL1 and HYAL2 inhibit invasion and migration of colorectal cancer cells through downregulation of MMP2 and MMP9 and concurrent upregulation TIMP2 and TIMP1 levels [210]. Decreased HYAL1 expression also correlates with early disease recurrence in endometrial carcinoma, larger tumor sizes and lymphovascular invasion. In addition, the reduced HYAL1 expression is associated with the depletion of E-cadherin, hinting at invading tumor cells undergoing EMT [251].

The notion that HYALs are overexpressed in some malignancies and downregulated in others, lead to their classification as either tumor promoting or suppressing effectors. Lokeshwar et al. showed that HYAL1 in prostate cancer can function as either on a concentration-dependent manner [252]. This, in addition to the tight regulatory network of HA synthases, HYALs and HA molecular sizes creates an intricate control in the tumor microenvironment.

#### 5.2.2. TMEM2 and CEMIP in Malignancies

In contrast with the ambivalent role of the traditional HYAL members, the more recently characterized HA-degrading TMEM2 and CEMIP appear to be involved in tumor progression in a more straightforward manner. Overexpression of either TMEM2 or CEMIP is a poor prognosis indicator in pancreatic ductal adenocarcinoma and siRNA knockdown of CEMIP expression leads to a decrease in cell migratory capacity. Interestingly, however, TMEM2 inhibition has the opposite effect on migration; the observed effect is explained by the concurrent increase in CEMIP levels, suggesting an interaction between the two in HA degradation control [215,253]. Moreover, TMEM2 is shown to mediate migration and metastasis in breast cancer through direct activation by SOX4 [214].

CEMIP is induced in colon cancer cells and its overexpression correlates with poor survival and its knockout leads to tumor growth attenuation and increased HA deposition [254]. Additionally, it is shown to promote ovarian cancer growth and progression by activating the PI3K/AKT signaling pathway, while its knockdown leads to attenuation of cell migration and invasion through the decrease in MMP2 and VEGF-A levels [217]. Further highlighting its role in tumor metastasis, CEMIP has been implicated in EMT and increased cancer cell migration [255,256]. Finally, CEMIP is enriched in brain metastatic exosomes and predicts disease progression and patient survival, with its function involved in inducing a pro-inflammatory state in the brain microenvironment and forming a pre-metastatic niche [216].

#### 5.2.3. Hyaluronidases in Cancer Therapy

Taking into account the involvement of HYALs in tumor progression, their targeting could become the basis for the development of an effective anti-cancer approach. O-sulfated HA (sHA) has previously been reported as a potential inhibitor of HYALs resulting from both competitive but mainly uncompetitive mechanisms [257]. Its biological activity has been tested in prostate cancer cells where it inhibits the activity of HYAL1, causing significant decrease in the proliferative and invasive cell capacities. In addition, sHA inhibits the PI3K/AKT signaling pathway and correlates with reduced HA receptor expression, suggesting a feedback loop between HA degradation and signaling in tumor cells [258]. Furthermore, a possible antitumor effect of sHA has also been studied in pre-clinical models of bladder cancer, where sHA fragments significantly attenuate the proliferation, migration and invasion of HYAL1-expressing cancer cells, while also inhibiting angiogenesis [259].

Apart from their inhibition, HYALs themselves can be employed as a potential strategy against cancer. Tumors are often characterized by accumulation of ECM components on their microenvironment, contributing to chemoresistance. HA is highly concentrated in pancreatic ductal adenocarcinoma and is associated with poor tumor prognosis and high interstitial fluid pressure. A recombinant human HYAL, PEGylated human hyaluronidase PH-20 (PEGPH20) has been used in this malignancy alongside cytotoxic agents to enhance their delivery and potency by degrading the HA surrounding the tumor stroma [260]. The promising results generated in preclinical models lead to the conduction of several phase I, II and III clinical trials for PEGPH20 in pancreatic cancer patients [261]. While the prospect for HYAL utilization seems beneficial, adverse effects from residual HA fragments could be a cause for concern as they can negatively affect tumor cell proliferation, migration and angiogenesis.

## 6. Conclusions

The complex network of ECM remodeling enzymes rules vital processes of cancer cells, such as proliferation, motility, EMT, invasion, autophagy and angiogenesis, contributing tumor aggressiveness and metastatic potential of several malignancies. MMPs, plasminogen activators and cathepsins master ECM proteolysis, triggering cancer cell invasiveness and the establishment of distant metastases. Glycolytic enzymes such as HPSE and HYALs, demonstrate unique roles in tumor progression, through HS and HA cleavage, respectively, thus modulating invasive and angiogenic events. In many cancer cases, the enzymatic activity of the matrix partners and EVs release are elevated, thus affecting cell-cell communication and subsequent signaling pathways in recipient cells, while often being correlated with aggressive tumor phenotypes. Another mechanism by which the ECM remodeling enzymes guide tumor progression is the epigenetic regulation of miRNAs that modulate the subsequent mRNA targets.

Considering the established roles of matrix enzymes in cancer development and metastasis, it is sensible to view them as attractive pharmacological targets for the development of cancer therapies (Table 4). The role of matrix remodeling enzymes is currently under investigation in ongoing clinical trials both in adult and children cancer types, such as leukemia and lymphomas, where the underlying mechanisms may offer new tools for the disease treatment. Novel strategies for highly selective pharmacological targeting of key ECM enzymatic partners may offer new prospects for studying complex processes of matrix reorganization and will contribute in designing effective, personalized therapeutic approaches for adult and children cancer management.

## Figures and Tables

**Table 1:** The main functions of major MMPs and TIMPs in cancer progression. Their prognostic value has been estimated by pan-cancer analysis in all available normal and tumor RNA Seq data from GEO, TCGA and GTex databases [59]. Significant differences of expression in tumor tissues compared to normal expression are marked with red (upregulation) and blue (downregulation) and have been computed by Mann–Whitney U test.

Enzyme	Main Functions	PanCancer Meta-Analysis	References
MMP1	Promotes pulmonary adenocarcinoma, mammary ductal carcinoma and pancreatic cancer; increased invasion in melanoma; promotes cell proliferation and differentiation of hepatocellular carcinoma cells; anti-angiogenic properties	Bladder; breast; colon; esophageal; lung; pancreas; rectum; hepatocellular carcinoma	[37,48,50,58]
MMP2	Increased migration invasion and metastasis; poor prognosis for breast, hepatocellular cancer; collagenolytic pathway driver for lymphatic vessel formation	Bladder; breast; colon; lung; ovary; pancreas; prostate	[6,60]
MMP3	Poor survival for pancreatic, pulmonary, breast carcinomas	Colon; esophageal; rectum; skin; prostate; stomach	[37]
MMP7	Poor prognosis in colorectal tumors; brings antiapoptotic and chemoresistance signals to colon cancer cells; promotes EMT	Pancreas; ovary; prostate; renal; skin; uterus	[6,61]
MMP9	Basement membrane destruction supports increased invasiveness, spreading, angiogenesis of various cancer types (melanoma, colorectal, lung, breast, ovarian cancer)	Acute myeloid leukemia (AML); breast; colon; lung; pancreas; testis; thyroid; uterus; skin	[6,36]
MMP10	Positively correlated with the invasiveness of human cervical, gastric and bladder cancers	Bladder; lung; esophageal; pancreas; breast; prostate	[62]
MMP11	Promotes cancer development (gastric, breast, pancreatic) by inhibiting apoptosis and enhancing migration and invasion; negative role against cancer development via suppressing metastasis in animal models	Bladder; breast; colon; esophageal; lung; ovary; pancreas; rectum; stomach; uterus; skin	[63]
MMP13	Promotes nasopharyngeal carcinoma metastasis; promotes angiogenesis in head and neck squamous cell carcinoma	Bladder; breast; esophageal; lung; ovary; skin	[64,65]
MMP14	Modulates melanoma cell dissemination and metastasis; drives breast cancer cell invasion through force-producing proteolytic contacts	Adrenal; bladder; breast; colon; liver; lung; ovary; pancreas; prostate; renal; skin; testis; uterus	[31,47]
MMP15	Promotes angiogenesis; drives EMT in lung, ovarian and colon cancer cells	Adrenal; bladder; esophageal; lung; ovary; pancreas; rectum; skin; testis; thyroid; uterus	[66]
MMP16	Promotes invasion and metastasis in melanoma and pancreatic cancer	Adrenal; bladder; breast; colon; esophageal; ovary; pancreas; prostate; skin; thyroid	[67]
MMP17	Induces angiogenesis promote growth and metastasis	Ovary; prostate; rectum; skin	[24]
MMP19	Modulates proliferation, adhesion, and metastasis in non-small cell lung carcinoma	Adrenal; breast; colon; lung; ovary; skin; pancreas; prostate; thyroid; uterus	[68]
MMP24	Progression in brain tumors, aides in migration and metastasis	AML; breast; ovary; renal; skin; thyroid; testis	[69]
MMP25	Promotes colon cancer growth	AML; lung; breast; ovary; pancreas; renal; skin; testis; thyroid	[70]
MMP26	Promotes glioma and non-small cell lung cancer invasion and metastasis	AML; prostate; brain; renal; testis; uterus; lung	[71,72]
TIMP1	Anti-apoptotic activity and anoikis resistance; promotes tumor progression in melanoma, malignant non-Hodgkin’s lymphomas and colon cancer	Adrenal; AML; breast; colon; esophageal; pancreas; prostate; rectum; skin; stomach; thyroid; uterus	[6,73,74]
TIMP2	Impairment of pro-MMP2 activation by MMP14; inhibits tumor growth and angiogenesis; anti-apoptotic activity	Adrenal; bladder; breast; colon; lung; ovary; pancreas; prostate; rectum; skin; testis; thyroid; uterus	[6,73,74]

**Table 2:** The main functions of plasminogen activation system components and cathepsins in cancer progression. Their prognostic value has been estimated by pan-cancer analysis in all available normal and tumor RNA Seq data from GEO, TCGA and GTex databases [59]. Significant differences of expression in tumor tissues compared to normal expression are marked with red (upregulation) and blue (downregulation) and have been computed by Mann–Whitney U test.

Enzyme	Main Functions	PanCancer Meta-Analysis	References
*Plasminogen activation system*			
Plasminogen/plasmin	ECM remodeling, activation of growth actors and enzymes, induction of migration, regulation of inflammation.	Adrenal; AML; breast; colon; lung; ovary; rectum; renal; skin; testis; thyroid	[153,154]
uPA	Associates with aggressive behavior. Contributes in cancer dissemination and metastasis via plasmin activation.	Adrenal; AML; bladder; breast; colon; esophageal; liver; lung; ovary; pancreas; prostate; rectum; renal; skin; stomach; testis; thyroid; uterus	[56,155]
tPA	Key role in fibrinolysis. Induces cell proliferation in pancreatic cancer. Relates to increased invasiveness, metastasis and poor prognosis in breast carcinomas. Shorter relapse-free and overall survival rates in colorectal cancer.	Adrenal; AML; colon; esophageal; liver; lung; ovary; pancreas; prostate; rectum; renal; skin; testis; thyroid; uterus	[156,157]
PAI-1	Poor prognosis in prostate cancer; mediates inflammation and pro-tumorigenic signals through tumor microenvironment; promotes metastasis	Breast; colon; esophageal; liver; ovary; Pancreas; prostate; renal; stomach; testis; thyroid; uterus	[135,142]
uPAR	Promotes aggressive cell behavior; invasion/metastasis, cell death resist, sustained proliferation in colorecteral cancer. Affects EMT and acquisition of breast cancer cell stem cell properties. Associates with higher grade tumors and recurrence.	AML; bladder; breast; colon; esophageal; liver;lung; ovary; pancreas; prostate; rectum; renal; skin; stomach; testis; thyroid; uterus	[138,140]
*Cathepsins*			
CTSB	Associates with tumor progression, higher metastatic burden in pancreatic and breast cancer. Targets E-cadherin to disrupt cell-cell junctions. Degrades matrix components to promote invasion.	Adrenal; AML; breast; colon; esophageal; liver; lung; ovary; pancreas; prostate; rectum; renal; skin; stomach; testis; thyroid; uterus	[158,159]
CTSC	Pro-angiogenic signaling, overt growth in squamous cell carcinoma.	Adrenal; AML; bladder; colon; esophageal; liver; lung; ovary; pancreas; rectum; skin; stomach; testis; thyroid; uterus	[160]
CTSD	High metastatic potential, low survival rates in breast cancer. Interferes with mTORC1 signaling to induce proliferation in breast cancer.	Adrenal; AML; breast; colon; esophageal; liver; lung; ovary; pancreas; prostate; rectum; renal; skin; stomach; testis; thyroid; uterus	[158,161]
CTSG	Increases MCF7 breast cancer cell aggregation. Overexpression correlates to acute lymphoid leukemia relapse.	AML; bladder; breast; colon; esophageal; liver; lung; ovary; pancreas; prostate; rectum; renal; skin; stomach; testis; thyroid; uterus	[162,163]
CTSK	Mediates tumor metastasis in colorectal cancer and skeletal metastasis in prostate cancer.	Adrenal; AML; bladder; breast; liver; lung; ovary; pancreas; prostate; stomach; testis; thyroid; uterus	[164,165]
CTSL	Expressed in advanced stage breast IDC. Upregulates EMT-related transcription factors in lung cancer. Sheds perlecan and E-cadherin.	Adrenal; AML; breast; colon; esophageal; liver; lung; ovary; pancreas; prostate; rectum; renal; skin; stomach; testis; uterus	[166,167]
CTSS	Contributes to cell proliferation, angiogenesis and tumor growth. Linked with lower recurrence-free survival rates in colorectal cancer.	AML; breast; colon; esophageal; liver; lung; ovary; pancreas; prostate; rectum; renal; skin; stomach; testis; thyroid; uterus	[168,169]
CTSV	Degrades elastin. Furthers cell proliferation and invasion, favors distant metastasis in breast cancer.	Adrenal; AML; bladder; breast; colon; esophageal; liver; lung; ovary; pancreas; prostate; rectum; renal; skin; stomach; testis; thyroid; uterus	[166,170]
CTSZ/X	Enhances cell motility and invasion in hepatocellular carcinoma. Upregulation linked with increased invasiveness in gastric cancer.	AML; bladder; breast; colon; esophageal; liver; lung; ovary; pancreas; prostate; rectum; renal; skin; stomach; testis; thyroid; uterus	[171,172]

**Table 3:** The main functions of major glycolytic enzymes in cancer progression. Their prognostic value has been estimated by pan-cancer analysis in all available normal and tumor RNA Seq data from GEO, TCGA and GTex databases [59]. Significant differences of expression in tumor tissues compared to normal expression are marked with red (upregulation) and blue (downregulation) and have been computed by Mann–Whitney U test.

Enzyme	Main Functions in Cancer	PanCancer Meta-Analysis	References
*Glycosidases*			
HPSE	Degrades basement membrane; stimulates expression of RANKL; stimulates exosome secretion; promotes angiogenesis; induces EMT; increases syndecan-1 shedding (myeloma) Poor prognosis marker in breast, ovary, pancreas, stomach, lung cancer and melanoma	adrenal; AML; breast; esophageal; lung; pancreas; rectum; renal; skin; stomach; testis; thyroid	[201,204,205,206,207] [194,196,197,198,199,200]
HYAL1	Promotes tumor growth and angiogenesis; it is repressed by Erα breast cancer Suppressive role in metastasis in colorectal cancer Exosomes containing HYAL1 stimulate migration in prostate cancer	adrenal; breast; liver; lung; pancreas; renal cell carcinoma; skin; stomach; thyroid; uterus	[208,209] [210] [211]
HYAL2	Antioncogenic activity in lymphoma Suppressive role in metastasis colorectal cancer	breast; lung; ovary; pancreas; rectum; testis;	[212] [210]
HYAL3	Predominant HYAL expressed in endometrial cancer	adrenal; AML; breast; colon; liver; ovary; pancreas; prostate; rectum; skin; testis; uterus	[213]
TMEM2	Mediates metastasis and invasion in breast cancer Poor prognosis marker for pancreatic ductal adenocarcinoma	AML; colon; esophageal; lung; pancreas; rectum; stomach; testis	[214] [215]
CEMIP	Elevated in exosomes driving brain metastasis Silencing decreases cell proliferation, migration and invasion in ovarian cancer)	colon; pancreas; rectum; stomach	[216] [217]

**Table 4:** Summary of major matrix enzymes as pharmacological targets, their mode of action and clinical development.

Target	Inhibitor	Mode of Action	Clinical Development
MMPs	Neovastat	MMP2/9/12 inhibition	Phase I/II/III
	BMS-272591	Broad-spectrum MMP inhibitor	Phase I/II/III
	Marimastat	Broad-spectrum MMP inhibitor	Phase I/II/III; discontinued
	Col-3 (metastat)	Selective inhibitor MMP2/9	Phase I/II
	Periostat	Broad-spectrum MMP inhibitor	FDA approved
uPA	Å6	uPAR antagonist	Phase I/II
	Upamostat	uPA inhibitor	FDA approved
Cathepsins	Odanacatib	Cathepsin K inhibitor	Phase III; discontinued
Heparanase	PI-88 (muparfostat)	HPSE inhibitor	Phase I/II/III
	PG545 (pixatimod)	HPSE inhibitor	Phase I
	SST0001 (roneparstat)	HPSE inhibitor	Phase I
	M402 (necuparanib)	HPSE inhibitor	Phase I/II
HYALs	PEGPH20	Degradation of HA in the surrounding tumor stroma	Phase I/II/III

## Data Availability

Data sharing not applicable.
